# Migratory Fish Bone in the Thyroid Gland: Case Report and Literature Review

**DOI:** 10.1155/2018/7345723

**Published:** 2018-02-22

**Authors:** EnHao Wu, Lei Huang, Ya Zhou, Xun Zhu

**Affiliations:** Department of General Surgery, The Second Affiliated Hospital of Soochow University, Suzhou, Jiangsu 215004, China

## Abstract

**Introduction:**

Foreign body stuck in the throat is a common emergency case, which can be removed by the endoscopic treatment. Fish bones are one of the common observed foreign bodies in the pharynx or cervical esophagus. Fish bones have a risk of damaging the mucosa when lodged in the upper digestive tract. Foreign bodies of fish bones located outside the laryngopharyngeal tissue are relatively unusual, and it is even more rare that they remain in the thyroid. It may cause local infection, abscess formation, large blood vessels rupture, and other serious life-threatening complications when the position of the fish bone migrates to the neck. We present a unique case of a 31-year-old woman in whom a fish bone was found in the thyroid. The fish bone had been removed successfully two months after the onset of symptoms. The relevant literature is reviewed and summarized.

**Case Presentation:**

A foreign body which is located in the neck area by swallowing is usually found in the emergency case. One of the commonest foreign bodies is the fish bone. The common presenting symptoms include foreign body (FB) sensation and or a sharp pain during swallowing. But we report a rare case in which a migratory fish bone stuck in the thyroid gland was found after 3 months. We retrieved previous literature and made a summary.

**Conclusions:**

Fish bones are not easy to be found as a foreign body. Surgeons should be aware that fish bones can become lodged in the thyroid gland. Combined with the history should be a wary fish bone to migrate to the case of the thyroid, to avoid misdiagnosis. To confirm the diagnosis, we can take ultrasound, computerized tomographic scanning (CT), and other tests.

## 1. Introduction

Foreign body stuck in the throat is a common emergency case which can be removed by the endoscopic treatment [1]. Fish bones are one of the common observed foreign bodies in the pharynx or cervical esophagus [2]. Fish bones have a risk of damaging the mucosa when lodged in the upper digestive tract. Foreign bodies of fish bones located outside the laryngopharyngeal tissue are relatively unusual and it is even more rare remain in the thyroid. It may cause local infection, abscess formation, large blood vessels rupture, and other serious life-threatening complications when the position of the fish bone migrates to the neck. We present a unique case of a 31-year-old woman in whom a fish bone was found in the thyroid. The fish bone had been removed successfully two months after the onset of symptoms. The relevant literature is reviewed and summarized.

## 2. Case Presentation

A 31-year-old woman suffered from sudden onset pharyngeal pain after she had eta fish during a dinner party. At that time, she tried to swallow rice and vegetable roll in order to dislodge the fish bone when she felt the pain of the foreign body, but it was not effective. Subsequently, she underwent fiberoptic laryngoscopy because of the sensation of foreign body in the pharynx. But the result of examination showed that no foreign body and no abnormalities were detected. The uncomfortable symptoms were alleviated after a week and therefore no further diagnosis and treatment were done.

She occasionally suffered from the foreign body sensation and tingly sensation on the left neck area when turning her head within two months after onset of symptoms. She came to our hospital for further examination. Except for mild tenderness at the left neck, there are no other pathognomonic signs from the physical examination which are specifically related to a residual foreign body in the neck.

**Figure 1:**
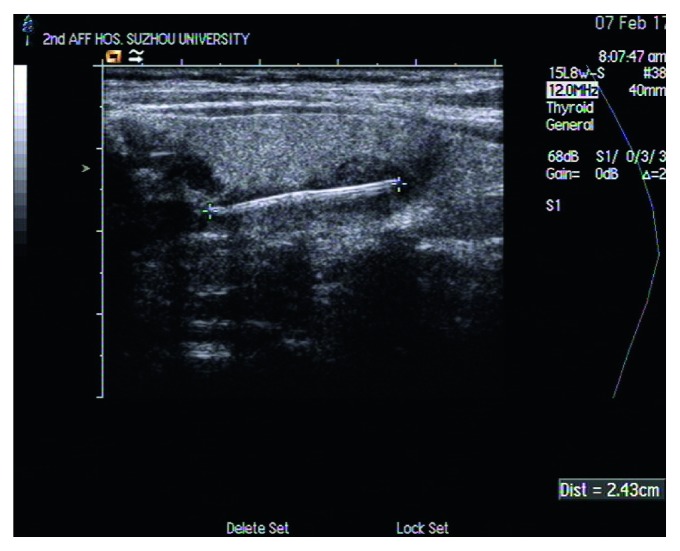
The features of ultrasonography. The presence of a foreign body that was approximately 2.43 cm hyperechoic linear image embedded in the left part of the thyroid lobe, and part of it was located outside the back membrane of the thyroid. A hypoechoci area surrounding the foreign body is regarded as the ultrasonic characteristics of inflammatory response.

After neck ultrasonography had been performed, the report revealed the presence of abnormal echo structure which was approximately 2.43 cm hyperechoic linear image embedded in the left part of the thyroid lobe and surrounded by a thin hypoechoic area (Figure 1). Other routine examination showed no abnormalities, including blood examinations and thyroid function tests. Combined with medical history, the diagnosis was residual fish bone in the thyroid.

**Figure 2:**
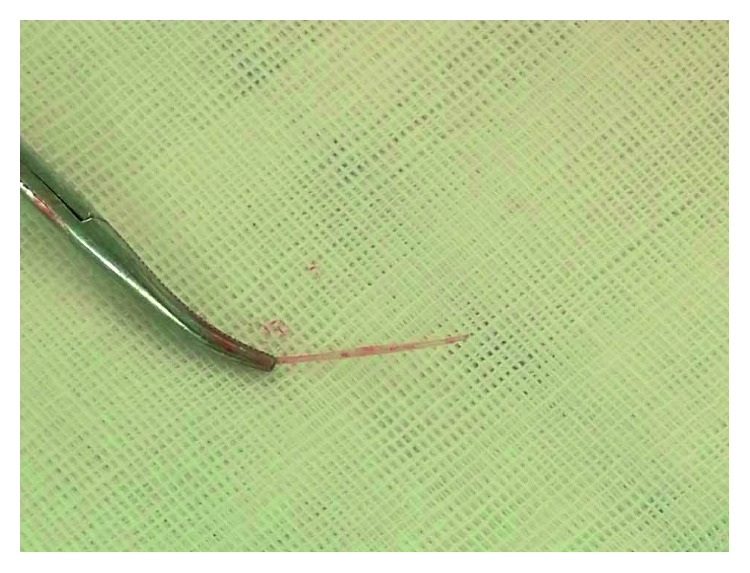
The picture of the fish bone. The fish bone with a sharply pointed tip was grasped and pulled out by the mosquito forceps. The length was about 2.45 cm.

**Figure 3:**
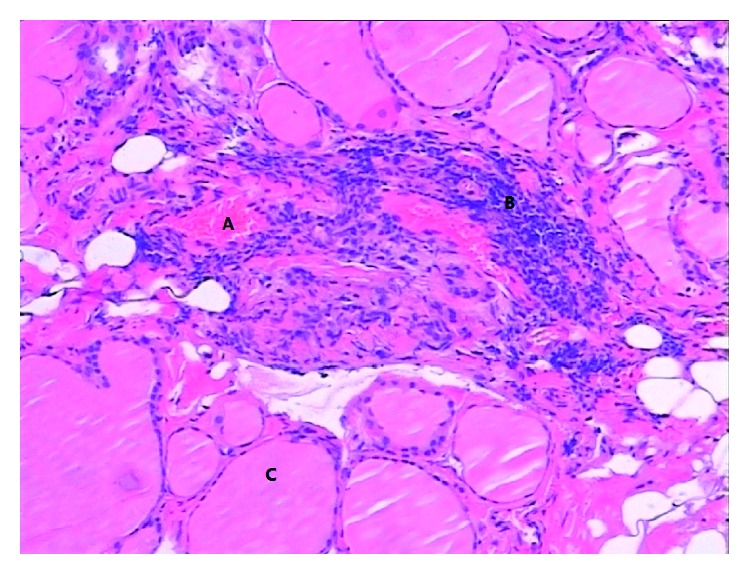
The appearance of histopathology (HE, ×100). Under the light microscope, the thyroid histopathological sections with HE staining. (A) The area of the hemorrhage cavity after pulling out the fish bone. (B) The well-defined region composed of macrophages and inflammatory cells in accordance with classic foreign body granulomatous inflammation. (C) Normal thyroid follicle.

In order to remove the foreign body, the patient underwent an exploratory surgery. After isolating and cutting the left middle thyroid vein, a sharp object piercing the surface of the dorsal thyroid membrane was found. We held the tip of the object with a mosquito forceps and pulled it out gently. The sharp object was a fish bone about 2.45 cm in length (Figure 2). Confirming that the esophagus, vascular, and nerve had not been damaged, left thyroid lobectomy had been performed. A drain was left for 24 hours. The postoperative course was uneventful, and the patient was discharged after 3 days. The pathological results showed that a well-defined region composed of macrophages and inflammatory cells around the hemorrhage cavity in accordance with classic foreign body granulomatous inflammation (Figure 3).

## 3. Literature Review

Many cases of foreign bodies embedded in upper digestive tract have been reported. But it is exceedingly rare that the fish bone penetrates through the esophagus wall and moves to the thyroid gland. We retrieved the PubMed database for a case report of the fish bone in the thyroid published in English from January 1910 to June 2017. The retrieval results included 16 articles and 18 cases. According to the PubMed database, the earliest reported case of a migratory fish bone in the thyroid was published in 1949 [3]. However, due to the long history, we could not obtain the original text and abstract of this case, and the clinical data comes from Hohman's literature [4]. We reviewed 17 cases reported in the literature and the present case (Table 1) [3, 5–19].

## 4. Discussion

Interestingly, all of the 18 patients with migratory fish bone in the thyroid gland were female with a median age of 59 years old (from 26 to 80 years old). Among them, 13 cases are greater than or equal to 50 years old. Except for 1 case in Europe, data of one case were not available, 16 of the remaining cases were in Asia. Among them, there were 5 cases in East Asia, 4 cases in West Asia, 6 cases in South-East Asia, one case in South Asia respectively. The geographical features of this disease may be related to the dietary habits of people in the area where persons more like to eat whole fish with bones as we know. Hohman et al. took into consideration that the elderly women with anodontia or dentures might have been at a higher risk, who are likely to swallow food whole without complete mastication and therefore less likely to detect the fish bone [4].

Fish bone impaction in the throat or esophagus is a common emergency. However, the fish bone that penetrates the cervical esophagus and migrates into the thyroid gland is relatively large, stiff, and sharp. Except for the 4 case reports that were not mentioned, 17 other cases reported a detailed description of the fish bone length, with a median length of 2.6 cm (from 2.0 to 4.1 cm). This size fish bone has thin sharp end and has enough hardness to penetrate the cervical esophagus wall. The fish bone migration to thyroid gland is also attributed to several other factors, such as the orientation of fish bone, contraction of the cricopharyngeus muscle during swallowing, contraction, and relaxation of the neck muscles during neck movement, and local inflammation of the esophageal or pharyngeal wall and direct pressure necrosis [5]. In all of 18 cases, 13 cases had the fish bone embedded in the left thyroid gland, while only 5 cases in the right thyroid gland. The cause of this probability of occurrence may be due to anatomical factors. The cervical esophagus is slightly deflected to the left side of the trachea, where a small part of the ventral side is not covered by trachea and close to the dorsal aspect of the left thyroid so that the fish bone can come directly through the esophagus wall to left thyroid.

The time from ingestion to presentation varied from a few hours to 9 months. We can see that most patients were presented within 24 h, but 9 of 18 cases had been diagnosed and treated for more than one week from ingestion. The reason for the delay of the disease is probably because (1) some older patients may be tolerant of pain or insensitivity to pain, (2) minor symptoms may have been ignored, and (3) initial examination negative results may mislead diagnosis. In the present case, the patient preferred to swallow something to dislodge the fish bone at the first time. When symptoms did not decrease, she underwent fiberoptic laryngoscopy, and the result was negative. She returned 2 months later after administering definitive treatment.

Most of the clinical manifestations are mainly foreign body sensation, pain, dysphagia, and neck mass. The most common symptom is pain caused by the foreign body damaging the mucosa. Sergi et al. described that when a fish bone penetrates the esophageal wall, the most common early symptoms are sudden pain and severe discomfort at rest. When the fish bone passes through the esophageal wall, symptoms soon become less evident, and the only clinical indicators are a persistent neck pain and a slight dysphagia [13]. These were similar to the present case. If the clinical symptoms are not obvious, the patients will not take care of it while the fish bone may remain for a long time. Long-term retention of foreign bodies can lead to chronic atypical symptoms, such as swallowing pain, dysphagia, swelling of the neck, neck mass, fever, severe systemic inflammatory response syndrome, and other clinical manifestations. In the present case, we failed to touch the mass, but there was inflammatory response around the fish bone in pathology.

The main methods of examination of foreign bodies in upper digestive tract include barium radiography, laryngoscope, X plain film, color Doppler ultrasound, CT, or MRI. Barium radiography is the first and most commonly used imaging method in the diagnosis of foreign bodies in the upper digestive tract. For this size of foreign bodies like a fish bone, the preferred way can be laryngoscopy or esophagoscopy (ESO). Laryngoscopy can be divided into direct laryngoscopy or indirect laryngoscopy. But these two methods of examination are most common in esophageal foreign body examinations. When the patients go to the hospital, most can be successfully treated. But if the foreign body is not found or found incomplete, there is a risk of a residual foreign body unknown. In the literatures, we found X plain film is a good way to find the foreign body. However, there is a high rate of missed diagnosis. A literature shows that physicians in the accident and emergency department and department of ENT (ear-nose-throat) had a higher rate of misdiagnosis by the X plain [20]. Ultrasonography is one of the few diagnostic modalities that can be done at the bedside and offers many advantages over other modalities. It is readily accessible and portable, and images are viewed in real time. Besides, it is less expensive and noninvasive than other modalities. They are not always useful but can be noninvasive and practical. CT examination can display the foreign body well, locate the foreign body accurately, and show the size, shape, position, direction, and the relationship of the foreign body with the surrounding tissue, and it can tell the extent of damage and the surrounding condition can be determined. CT is recommended in checking the foreign body, such as the fish bone [21]. In the present case, the color Doppler ultrasound has confirmed the result, so we did not perform the CT examination.

Most of foreign bodies trapped in the throat area can be treated by the endoscopic treatment [22]. However, the treatment of the patients in whom the fish bone stuck in the thyroid gland usually is hemithyroidectomy, because of inflammation and abscess surrounding the fish bone. U. D. Arumainathan et al. [14] reported the first case in which the fish bone lodged in the thyroid was removed without the need to remove the entire gland in 2000. In our case, thyroidectomy had been performed to remove the inflammatory granuloma in the left thyroid.

## 5. Conclusions

Fishbone is not easy to be found as a foreign body. Surgeons should be aware that fish bones can become lodged in the thyroid gland. Combined with the history should be a wary fish bone to migrate to the case of the thyroid, to avoid misdiagnosis occurred. To confirm the diagnosis, we can take ultrasound, CT, and other tests. For the treatment of fish bone in the thyroid, the first is to define the location of the lesion and the extent of the inflammatory response and finally to decide the way to effectively clear the lesions.

## Figures and Tables

**Table 1:** The clinical data, diagnosis, and examinations for reported cases.

Case	Year	Country	Male (M)/female (F)/age	Left (L)/right (R)	Disease of course	Length (cm)	Symptom and signs				The methods of examination				
							FBS	Pain	Dysphagia	Neck mass	X-ray	US	CT	FFL	ESO
1	2017	China	F (31)	L	2 months	2.45	(+)	(+)	(−)	(−)	−	+/+	−	+/−	−
2 [5]	2016	Malaysia	F (80)	L	6 days	4.0	(+)	(+)	(−)	(−)	+/+	−	+/+	+/−	+/−
3 [6]	2012	Japan	F (56)	L	2 months	2.8	(+)	(−)	(−)	(+)	−	−	+/+	+/−	+/−
4 [7]	2012	Japan	F (69)	R	9 months	3.4	(−)	(−)	(−)	(+)	−	+/+	+/+	−	−
5 [8, 9]	2010	Kuwait	F (28)	R	3 days	/	(−)	(+)	(−)	(+)	+	/	+/+	/	+/−
6 [8, 9]	2010	Kuwait	F (56)	L	2 months	/	(−)	(+)	(−)	(−)	+	/	+/+	—	+/−
7 [10]	2006	Pakistan	F (26)	R	3 weeks	/	/	(+)	/	/	+/+	/	+/+	+/−	/
8 [11]	2009	Taiwan	F (50)	R	1 month	4.1	(−)	(+)	(−)	(+)	+/+	−	+/+	+/−	−
9 [12]	2005	Japan	F (61)	L	Emergency	2.7	(−)	(+)	(−)	(−)	+/+	+/+	+/+	−	−
10 [13]	2002	Italy	F (59)	L	5 days	2.0	(+)	(+)	(−)	(−)	+/−	+/+	+/+	−	+/−
11 [14]	2000	Malaysia	F (38)	L	/	2.0	(−)	(−)	(+)	(−)	+/+	−	+/+	−	+/−
12 [15]	1999	Singapore	F (68)	R	4 days	3.0	(+)	(−)	(−)	(−)	+/+	−	+/+	−	+/−
13 [15]	1999	Singapore	F (65)	L	2 weeks	3.5	(+)	(−)	(−)	(−)	+/+	−	+/+	−	−
14 [16, 17]	1993, 1992	Israel	F (38)	L	5 days	2.5	/	(+)	/	(+)	+/+	+/+	/	+/−	/
15 [18]	1993	Singapore	F (72)	L	1 week	2.5	(+)	(+)	/	/	+/+	/	+/+	/	+
16 [18]	1993	Singapore	F (66)	L	3 days	2.5	(+)	(+)	/	/	+/−	+/+	/	+/−	+/−
17 [19]	1990	Kuwait	F (42)	L	Emergency	3.5	(−)	(+)	(+)	(−)	+/+	−	+/	+/−	+/−
18 [3]	1949	/	F (61)	L	3 weeks	/	/	/	/	/	+/+	/	/	/	/

FBS: foreign body sensation; FFL: flexible fiberoptic laryngoscopy; US: ultrasonography; ESO: esophagoscopy; /: not mentioned in the article; (+): positive; (−): negative; +/+: used/find; +/−: used/not find; −: not used.
